# Rocio Virus: An Updated View on an Elusive Flavivirus

**DOI:** 10.3390/v13112293

**Published:** 2021-11-16

**Authors:** Marielena Vogel Saivish, Vivaldo Gomes da Costa, Gabriela de Lima Menezes, Roosevelt Alves da Silva, Gislaine Celestino Dutra da Silva, Marcos Lázaro Moreli, Livia Sacchetto, Carolina Colombelli Pacca, Nikos Vasilakis, Maurício Lacerda Nogueira

**Affiliations:** 1 Laboratório de Pesquisas em Virologia, Departamento de Doenças Dermatológicas, Infecciosas e Parasitárias, Faculdade de Medicina de São José do Rio Preto, São José do Rio Preto 15090-000, SP, Brazil; gislaine.cds@gmail.com (G.C.D.d.S.); liviasacchetto@gmail.com (L.S.); 2 Instituto de Biociências, Letras e Ciências Exatas, Universidade Estadual Paulista (UNESP), São José do Rio Preto 15054-000, SP, Brazil; carolpacca@gmail.com; 3 Núcleo Colaborativo de Biosistemas, Universidade Federal de Jataí, Jataí 75801-615, GO, Brazil; gabrieladelima_@hotmail.com (G.d.L.M.); rooseveltfisicaufg@gmail.com (R.A.d.S.); marcos_moreli@ufg.br (M.L.M.); 4 Instituto Superior de Educação Ceres, Faculdade Faceres, São José do Rio Preto 15090-000, SP, Brazil; 5 Department of Pathology, University of Texas Medical Branch, 301 University Blvd., Galveston, TX 77555-0609, USA; 6 Sealy Center for Vector-Borne and Zoonotic Diseases, University of Texas Medical Branch, 301 University Blvd., Galveston, TX 77555-0609, USA; 7 Center for Biodefense and Emerging Infectious Diseases, University of Texas Medical Branch, 301 University Blvd., Galveston, TX 77555-0609, USA; 8 Center for Tropical Diseases, University of Texas Medical Branch, 301 University Blvd., Galveston, TX 77555-0609, USA; 9 Institute for Human Infection and Immunity, University of Texas Medical Branch, 301 University Blvd., Galveston, TX 77555-0610, USA

**Keywords:** Rocio virus, transmission cycles, epidemiology, pathogenesis, clinical manifestations

## Abstract

Rocio virus (ROCV) is a mosquito-borne flavivirus and human pathogen. The virus is indigenous to Brazil and was first detected in 1975 in the Sao Paulo State, and over a period of two years was responsible for several epidemics of meningoencephalitis in coastal communities leading to over 100 deaths. The vast majority of ROCV infections are believed to be subclinical and clinical manifestations can range from uncomplicated fever to fatal meningoencephalitis. Birds are the natural reservoir and amplification hosts and ROCV is maintained in nature in a mosquito-bird-mosquito transmission cycle, primarily involving *Psorophora ferox* mosquitoes. While ROCV has remained mostly undetected since 1976, in 2011 it re-emerged in Goiás State causing a limited outbreak. Control of ROCV outbreaks depends on sustainable vector control measures and public education. To date there is no specific treatment or licensed vaccine available. Here we provide an overview of the ecology, transmission cycles, epidemiology, pathogenesis, and treatment options, aiming to improve our ability to understand, predict, and ideally avert further ROCV emergence.

## 1. Introduction

Rocio virus (ROCV) is an arthropod-borne virus (arbovirus) in the genus *Flavivirus* (family *Flaviviridae*). As with other members of the genus *Flavivirus*, ROCV has a linear, positive-sense single-stranded RNA genome, approximately 11 kilobases in length encapsulated in virions of 43 nm in diameter [1]. It is the etiological agent of a potentially fatal neurological infection affecting humans in Brazil. The virus was first identified in the 1970s [2], during a series of outbreaks in coastal communities in the State of Sao Paulo resulting in the largest meningoencephalitis epidemic, causing fear and panic among the population [2,3]. Until then only two neurotropic flaviviruses, Saint Louis encephalitis virus (SLEV) and Ilheus virus (ILHV), were known to circulate in South America, causing sporadic human cases [4,5]. The first cases of meningoencephalitis were reported in April 1975, in several areas located on the coast of Sao Paulo State, and over the following months more than 1000 cases of encephalitis were reported, with a fatality rate of 13% as well as development of permanent severe neurological sequelae in 20% of the survivors [3,6]. However, a retrospective serosurvey suggested that the onset of the epidemic probably occurred earlier, between 1973–1974 [7]. The first virus isolate (strain SPH 34675) was isolated from suspensions of cerebellum and spinal cord from a fatal case of a male patient in December 1975, months after the onset of the outbreak, and the virus was named after the neighborhood of Rocio, located in the city of Iguape [2]. Nine ROCV strains were also recovered from CNS tissues of 17 other patients who died with encephalitis [2], sentinel mice [2], an Andean sparrow (*Zonotrichia capensis*) [2] a pool of *Psorofora ferox* [8], and partial virus fragments were detected bio-banked human sera collected during an outbreak of dengue fever in Goiânia in 2011 [9]. Subsequent serosurveys detected the presence of ROCV-specific antibodies in migratory birds (e.g., double-collared seedeater (*Sporophila caerulescens*) and creamy-bellied thrush (*Turdus amaurochalinus*)) [10,11], horses [12], water buffaloes (*Bubalus bubalis*) [13] and humans [14,15], suggesting undetected ROCV circulation in large geographic areas outside the State of Sao Paulo [9]. Interestingly, attempts at the time to better understand the epidemiology of encephalitis in patients with a history of febrile illness [7] were overshadowed by a widespread epidemic of meningococcal meningitis affecting the state of Sao Paulo, resulting in their misdiagnosis (reviewed in [16]). These observations highlight the emergence potential of ROCV to cause outbreaks and epidemics but also diagnostic challenges in accurately accessing the true burden of ROCV disease in the human population. In this review, we summarize our current understanding of ROCV’s host range, transmission cycles, epidemiology, pathogenesis and clinical outcomes of infection.

## 2. Genome Organization and Replication

**Figure 1:**
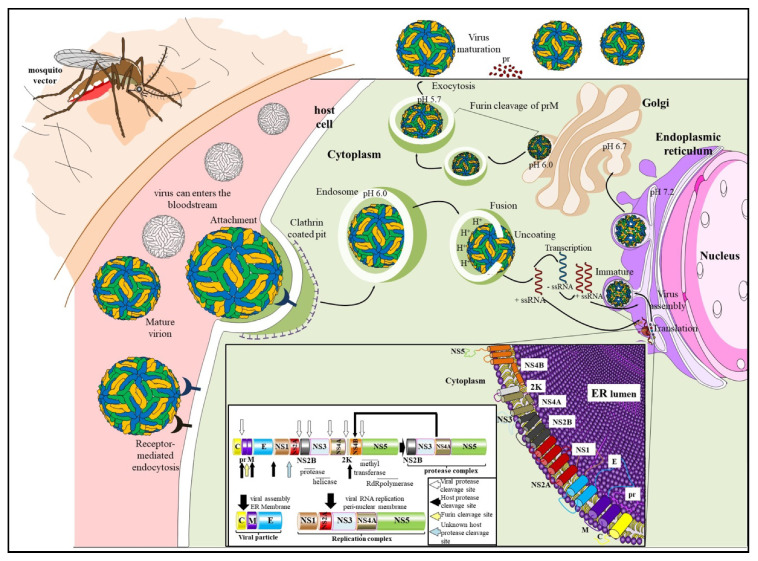
The replication cycle and genome organization of ROCV. The insert in the lower part of the figure shows the genome organization with the cleavage sites of the host and viral proteases of ROCV and the viral polyprotein at the endoplasmic reticulum membrane.

To date Tanaka et al. [1] remains the only study to have examined the replication of ROCV by electron microscopy, demonstrating that ROCV particles share the morphological characteristics common to members of the genus *Flavivirus*; spherical particles of 43 nm in diameter having an electron dense core surrounded by a host-derived lipid bilayer membrane [1]. The positive polarity single stranded flavivirus RNA genome is approximately 10.7 kb in length, encodes a single open reading frame (ORF), flanked by a type 1 capped 5′-terminal non-coding region (NCR), and a 3′-terminal NCR and lacking a poly-adenylation site [17,18]. The single ORF encodes three structural and seven nonstructural (NS) proteins, flanked by short non-coding regions: three structural proteins—capsid pro-tein (C), pre-membrane/membrane protein (prM/M) (formed by cleavage from its precursor prM) and envelope glycoprotein (E)—and seven non-structural proteins—NS1, NS2A, NS2B, NS3, NS4A, NS4B and NS5 (Figure 1, insert).

Details of the ROCV replication cycle are limited [1], but the replication cycle of members of the genus *Flavivirus* begins with the binding to host cell receptors and entering the cell through clathrin-mediated endocytosis. Following trafficking through endosomal compartments, envelope protein-mediated fusion of viral and cellular membranes, and dis-assembly of the virus particles, single-stranded viral RNA is released into the cytoplasm where translation occurs. Translation of the viral RNA into a polyprotein occurs at the ribosomes, which is further processed by host and viral proteases to non-structural (NS) and structural proteins, forming the replication complex within the endoplasmic reticulum (ER) to replicate the viral RNA. Viral particle assembly occurs on the membrane of the ER and particles bud into the ER as immature virus particles [19]. During egress of the progeny virus particle through the secretory pathway, pre-membrane (prM) protein is proteolytically cleaved by a host encoded furin protease [20] and mature virus particles are released into the extracellular space (reviewed in [21]) (Figure 1).

## 3. Transmission Cycles and Host Range

**Figure 2:**
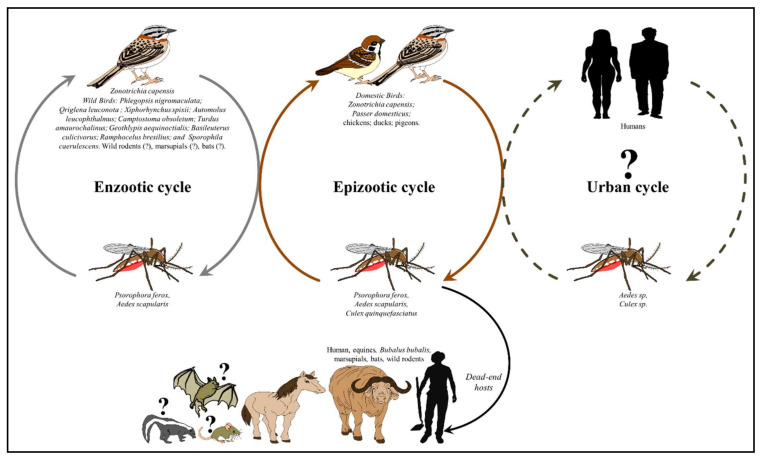
The transmission cycles of ROCV.

To date the transmission cycle of ROCV is not well understood. Based on surveillance and experimental studies it is likely that ROCV is maintained in nature in a mosquito–bird–mosquito transmission cycle, where humans are incidental dead-end hosts [8,16] (Figure 2). Comprehensive entomological surveillance during the outbreak of 1975–1976, implicated *Ps. ferox* as a transmission vector with the isolation of ROCV [8]. Interestingly in the study of Lopes et al [8], two of the *Ps. ferox* mosquitoes were engorged with canine blood. *Ps. ferox* is a floodwater mosquito species native to most of North and South America, typically found in woodland environments with pools that intermittently fill with rain or flood water [22,23]. It is a strong flyer with a long dispersal range, as far as 11 km from the point of release [24]. It is a competent virus of transmission for several arboviruses, including St. Louis encephalitis [25], Una [26], Ilheus [26] and Venezuelan equine encephalitis [27] and exhibits opportunistic feeding behavior, although mammalian blood sources are preferable to avian [28,29]. Coupled with the mosquito’s aggressive feeding behavior, this highlights its potential threat for public health.

**Figure 3:**
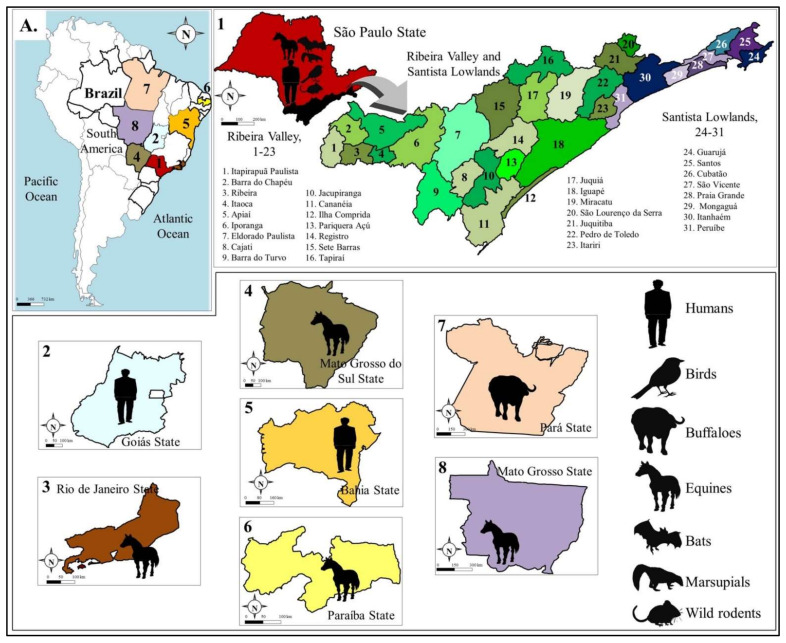
Overview of detected circulation of the Rocio virus in Brazil. (**A**). Brazilian states where detection of ROCV was documented. (**A1**) São Paulo State. During the outbreak of the 1970s, ROCV circulation was detected in 8 regions in the Santista Lowlands and 23 regions in the Ribeira valley. Serology showed flavivirus exposure in rodents, bats, marsupials, and pigeons; (**A2**) Goiás State; (**A3**) Rio de Janeiro State. Serologic detection in equines; (**A4**). Mato Grosso do Sul State. Serologic detection in equines; (**A5**) Serologic detection in humans; (**A6**) Paraíba State. Serologic detection in equines. (**A7**) Pará State. Serologic detection in buffaloes (*Bubalus bubalis*). (**A8**) Mato Grosso State. Serologic detection in equines.

The studies of Forattini et al [30,31,32] on culicine mosquitos in the Ribeira valley region in the state of Sao Paulo were seminal in shedding light on ROCV transmission cycles, by mapping the distribution and biting activity of *Psorophora, Aedes* and *Culex (Melanoconion*) species at an ecologic gradient, spanning sylvatic, transition and peri-domestic ecotypes (Figure 3). At the same time Mitchell et al were able to demonstrate the transmission potential of *Ps. ferox* and *Ae. scapularis* with experimental infection on viremic chickens [33,34]. Like *Ps. ferox*, *Aedes scapularis* mosquitoes have an exceptionally broad distribution, occurring from the Rio Grande Valley and the Florida Keys of the southern Unites States down to central Argentina, and throughout the Caribbean, except Puerto Rico [35,36]. It is an important mosquito vector competent for transmitting a diverse number of arboviruses, including yellow fever [37,38], Ilhéus [34,39] and Venezuelan equine encephalitis virus [5,40,41]. It is a generalist in its use of habitats (both sylvatic and peri-domestic), whose larval habitats are commonly found in temporary pools filled by rain or floodwater. Adults exhibit an opportunistic feeding behavior, preferring to feed in endothermic hosts [42,43,44,45], as well as synanthropic behavior, such as entering human dwellings and blood-feeding on humans indoors [46]. Collectively these studies establish the critical role played by *Aedes scapularis* as possible bridge vector between sylvatic and peri-domestic environments.

The virus was isolated from rufous-collared sparrows (*Zonotrichia capensis*) [2] in Sete Barras, a region that at the time was covered by primary forest. Rufous-collared sparrows have the largest distribution of any Neotropical passerines, ranging across southern Mexico, Central America, several Caribbean Islands and nearly the entire South America [47,48] and their migratory habits exhibit significant variations ranging, from sedentary to altitudinal to long distance latitudinal migrations [49]. An additional survey of wild birds (58 species belonging in 51 genera) at the time of the 1975 epidemic in the coastal counties of Peruibe and Itanhaem in 1975 showed nearly 25% seropositivity to flaviviruses, whereas seropositivity in wild (rodents, bats, marsupials, and pigeons) and domestic (chickens and ducks) animals ranged from 7.3–60% [3] (Figure 3). A subsequent longitudinal survey of wild birds from 1978 to 1990 in the counties of Salesopolis, Itapetininga and Ribeira Valley demonstrated monotypic seropositivity in 9/26,765 sampled birds, representing eight species of which two considered migratory (*Sporophila caerulescens* and *Turdus amaurochalinus*) [11] (Figure 3). These observations suggest that ROCV circulates among birds in the forest, brush and peri-domestic habitats in the state of Sao Paulo and that migratory birds may disperse the virus throughout the state or to other Brazilian states or countries along migratory routes. Experimental studies on house sparrows (*Passer domesticus*), a common bird throughout the Americas, demonstrated their susceptibility to ROCV infection, with viremia peaking at 4.3 log_10_/mL and duration of 2–3 days, suggesting its low potential for ROCV transmission [50]. One significant observation of this study was the heterotypic protection afforded by prior infection with SLEV, an enzootic virus in Brazil, against challenge with ROCV, which could play a role in determining ROCV distribution and transmission given the well-documented cross-protection between flaviviruses of the same antigenic complex [51,52].

More recent serological studies have also demonstrated evidence of ROCV infection in equines [12] and water buffaloes [13] in rural regions across Brazil. Surprisingly, monotypic exposure to ROCV was significantly high at 6.1% (46/753), whereas in water buffaloes this was 0.3% (2/654) of animals sampled (Figure 3). These animals are exposed to thousands of mosquito bites in places that may serve as transmission foci for ROCV. The high levels of ROCV seropositivity in horses, large domesticated animals living in close proximity to humans, combined with the lack of epizootic reports by veterinarians, strongly suggests the presence of asymptomatic or subclinical infections and should serve as a warning to the public health authorities regarding increased surveillance. It is also raising questions about their possible role as hosts in ROCV transmission. Naturally, the role of any vertebrate host in viral transmission must be assessed not only by their response to infection, but also by the relative efficiency of the vector and their respective densities. Therefore, a biologically poor vertebrate host(s) (e.g., house sparrows) could become epidemiologically important in areas with high population densities and presence of efficient vectors with low thresholds of infection (e.g., *Ps. ferox* or *Ae. scapularis*). Collectively, the transmission cycles of ROCV are not well understood and humans, horses and other vertebrates may serve as incidental dead-end hosts.

## 4. Human Epidemiology

The human epidemiology remains largely unknown, and the reasons of the virus periodic emergence remain a mystery. To date all the detected cases of ROCV in humans have been reported in three states (Table 1), although serologic evidence suggests that ROCV geographic range may extend to the state of Amazonas (see section above). Between March and June 1975 8 cities in the Santista Lowlands (Baixada Santista) and 23 cities in the Ribeira Valley, both in the state of Sao Paulo, reported 465 cases of febrile illness associated with encephalitis, with 61 deaths [3]. At the time of the outbreak, these regions were considered poor, with low per capita income, derived from the cultivation of bananas and tea. The region remains to date highly forested with currently approximately 14.4% of land cover is used for agricultural cultivation and cattle pasture (http://arquivo.ambiente.sp.gov.br/cpla/2018/05/proposta_zee_-valedoribeira_2014.pdf, accessed on 5 March 2021) and the rest is designated as a protected ecologic area (Atlantic rainforest). Most of the population (65.8%) lived in rural areas and 65.2% of the economically active population worked in rural activities (e.g., cultivation and ranching). The study showed an increased prevalence to flavivirus-specific antibodies [53,54] from previous years in the population, with males working in close contact with forested areas having a higher attack rate than females, suggesting that exposure to ROCV was more likely to occur away from home [3]. Critically, the study also demonstrated no evidence of person-to-person transmission in ROCV spread, reinforcing the notion of spread by hematophagous insects and that humans serve as incidental dead-end hosts. Subsequent serology surveys in the Ribeira Valley consistently demonstrated high prevalence of infection to arboviruses, with prevalence progressively increasing with age. Interestingly, ROCV prevalence was high in fishermen with two of showing evidence of recent infection, as well as in children living in coastal cities, suggesting urban transmission of ROCV [55,56,57].

Subsequent to the 1975–1977 outbreak, there was a precipitous decrease in the number of cases [58], with two IgM positive cases in school children reported in 1987 [15] and 6 IgG positive cases in residents at the Juréia-Itatins Ecological Station in 1990 [59] (Table 1). Interestingly, in a 1984 arbovirus serosurvey of 288 rural inhabitants in the state of Bahia, 4.3% were positive to arbovirus exposure, with a 12 year-old schoolgirl positive to ROCV with no previous travel history outside the state [60]. In 1995, three ROCV positive IgM cases were identified in Salvador, state of Bahia, during a dengue epidemic, which were initially misidentified as dengue infections, while a contemporaneous serologic survey of 689 serum samples collected in the cities of Ipupiara and Prado identified five additional patients positive for exposure to ROCV [14]. These were the first detections of the virus in the northeastern region of the country (Table 1), documenting the active circulation of ROCV outside the state of Sao Paulo. The virus remained undetected for nearly 17 years, until a recent retrospective serologic survey of 647 serum samples collected during a DENV epidemic in the state of Goias retrieved partial ROCV genetic sequences from two samples [9]. The patient samples had originally been misclassified as DENV, but further examination by molecular methods clearly identified them as ROCV. Review of the clinical records indicated non-specific febrile illness, presented with fever, myalgia, and arthralgia, and both reported no recent travel history [9].

Collectively, the data suggest an extended range of ROCV transmission in Brazil and reinforce the urgent need for improved diagnostic capabilities as well as sustainable arbovirus surveillance networks for the early and accurate detection of arboviral outbreaks.

## 5. Clinical Presentation and Pathogenesis of ROCV Infection

**Figure 4:**
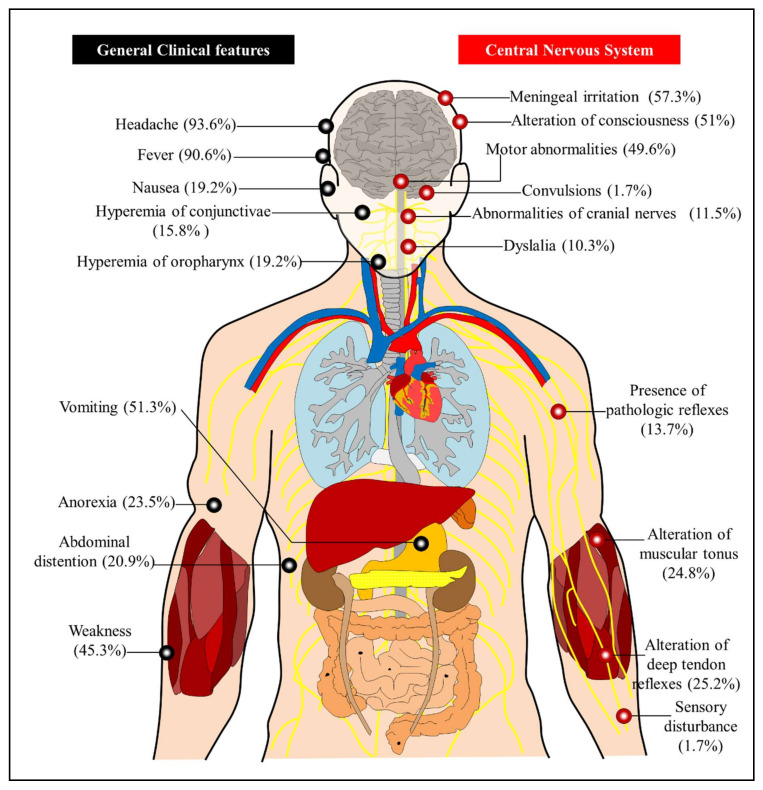
Clinical features of patients following ROCV infection.

Clinical manifestations of infection include fever, malaise, severe headache, lower-extremity weakness, and severe neurologic disorders, with permanent sequelae observed in 20% of surviving patients [2,7,9,15,61]. The clinical features of ROCV infection are depicted in Figure 4. The incubation period lasts 7 to 14 days, with patients presenting with abrupt onset of fever, severe headache, anorexia, nausea, vomiting, myalgia, and malaise, and in severe cases stupor and coma with respiratory complications [3]. Late signs of encephalitis included confusion, reflex disorders, motor impairment, meningeal irritation, cerebellar syndrome, and seizures. Neuropsychiatric sequelae were observed in 20% of the patients: visual, olfactory, and hearing disorders, lack of motor coordination, disturbance of balance, difficulty in swallowing, urinary incontinence and memory defects [3]. ROCV infections were lethal in 10% of cases. Autopsies of fatal encephalitis cases showed histopathological lesions in the brain and signs of thalamic inflammation [61].

The pathogenesis of ROCV infection is not fully understood. Within the genus *Flavivirus*, ROCV has been classified within the Japanese encephalitis virus (JEV) serogroup, whose members include St. Louis encephalitis virus (SLEV) and Ilhéus virus (ILHV). To date several rodent (BALB/c mice, C57BL/6 mice and golden hamster (*Mesocricetus auratus*)) studies have been performed, seeking to elucidate ROCV pathogenesis [1,62,63,64,65,66]. Electron microscopy of ROCV-infected suckling swiss mice brain tissues showed ROCV exclusively located in the lumen of membranous cytoplasmic organelles, principally within hypertrophic Golgi membranes and endo-plasmatic reticulum, intracytoplasmic vesicles, and the nuclear envelope [1], observations commonly seen in neurotropic flavivirus infections [67,68,69]. Virions were found in rows within organelles, but individually scattered particles were also common. Virions were absent in tissues collected prior to 40 h post infection (h.p.i.), and virus accumulation in infected sections increased rapidly in tissues collected subsequently, reaching a maximum in tissues collected from moribund animals of 73 h.p.i. Inclusion bodies consisting of electron dense masses were found in the cytoplasm of infected cells and were likely to be associated with virus replication [1].

ROCV-infected young adult BALB/c mice developed meningo-encephalomyelitis presenting with hind limb paralysis, muscle weakness, tremors, loss of balance and death 9 days post infection (d.p.i.) [63]. Significant amounts of inflammatory infiltrates (lymphocytes, NK cells, neutrophils, monocytes and macrophages) were observed starting at 4 d.p.i in the spinal cord and peaking at 9 d.p.i. in the brain, coinciding with the presence of the virus and development of acute flaccid limb paralysis and death, respectively. Immunohistochemistry showed viral antigen expression in neurons, astrocytes, microglia, endothelium, and macrophages in the spinal cord at 4 d.p.i. and in the brain at 8 d.p.i. Significant neuron degeneration was observed in the dentate gyrus and CA3 region of the hippocampus, likely to be due to ROCV infection and apoptosis induced by cytokines produced by glial and macrophage activated cells leading to necrosis and cell death, suggesting a critical role for macrophages in the pathogenesis of ROCV infection. [63]. Chavez et al further explored the immunopathogenesis of ROCV infection by demonstrating the significant role of the CC-chemokine receptor 5 (CCR5) and its interaction with the ligand macrophage inflammatory protein (MIP-1a). Using CCR5 and MIP-1a knockout mice they demonstrated increased survival rates compared to wild-type (*wt*) ROCV-infected animals, as well as significantly reduced inflammation and viral loads in the brains of the knockout mice, suggesting CCR5/MIP-1a mediates lymphocyte recruitment in the brain leading to disease severity [64]. Recently, Amarilla et al [66] investigated the role of monocytes in the ROCV-induced inflammatory response in the brain. Migration of monocytes into the brain is heavily dependent on the interaction between the C-C Motif Chemokine Ligand 2 (CCL2) and its receptor C-C Motif Chemokine Receptor 2 (CCR2). Using *wt* C57BL/6 mice they showed that ROCV infection induces the production of CCL2 in the blood and brain, resulting in the increased infiltration of macrophages and CD8+ T lymphocytes into the brain. Similarly, to the observations of de Barros et al [63], virus infiltration of the spinal cord occurred earlier than infiltration of the brain with the highest viral loads present in the cortex and hippocampus. This is in agreement with the histopathologic damage Rosemberg observed in the same regions of autopsied human brains infected with ROCV [61]. Conversely, the use of CCR2 knockout (*Ccr2^−/−^*) C57BL/6 mice, demonstrated that CCR2 is required for efficient infiltration of macrophages into the brain, which is associated with a reduction in disease severity and mortality [66].

Collectively, the rodent models described above demonstrate the neurotropic (spinal cord and brain) nature of ROCV infection, but they have not shed any light in the mechanism of neuro-invasion, reiterating the notion that ROCV pathogenesis remains poorly understood, and begging the urgent need of a suitable animal model mimicking human disease.

## 6. Diagnosis, Treatment and Prevention Options

The clinical presentation of ROCV infection is indistinguishable from the symptoms of other mosquito-borne flaviviruses, including neurotropic flaviviruses such as Japanese encephalitis, West Nile, and St Louis encephalitis viruses. As discussed earlier, ROCV infections have been often misdiagnosed with other arboviral infections endemic to Brazil, often as dengue or St. Louis encephalitis, therefore differential diagnosis of ROCV infection must be established in the laboratory. Diagnosis of infection with ROCV is possible with a variety of methods, including genetic (RT-PCR, qPCR) [70,71] and serologic (IgM and IgG ELISA, hemagglutination inhibition (HI) and plaque reduction neutralization test (PRNT)]). However, caution should be exercised in the utility of serologic tests in the differential diagnosis of ROCV, given the high level of antibody cross-reactivity among flaviviruses and the lack of routine laboratory serological assays that complicate accurate diagnosis of arboviruses, including ROCV. Therefore, there is an urgent need for the employment of new and affordable technologies [72,73] for the development of more accurate diagnostic assays.

There is no licensed vaccine or antiviral therapy available for ROCV infections, thus patient care protocols include stabilization and admission to the intensive care unit (ICU) for severe cases. Long term supportive care is recommended for survivors with long term sequalae. A three dose formalin inactivated vaccine developed in 1977 by Butantan elicited low rates of seroconversion in early phases of human trials and further development was discontinued [74]. Therefore, current efforts are centered around prevention strategies which are based on vector control strategies (door and window screens and elimination of breeding sites) and personal protection measures, such as protective clothing, use of insect repellents and behavior modification to minimize human contact at peak mosquito activity. Moreover, even after 50 years, ROCV’s transmission cycles are not well understood and thus any intervention will remain a formidable challenge. Complete eradication or control of the enzootic transmission cycle is nearly impossible since it is not amenable to typical control interventions (reviewed in [75]). Disrupting spillover into human agricultural and urban habitats may require novel approaches such as dynamic modelling and machine learning that take advantage of a multitude of available empirical data (e.g., host range, vectors of transmission and ecotypes) that have been acquired over time during documented ROCV spillover investigations [2,3,7,8,9,11,12,14,15,30,31,32,34,42,46,50,53,58,59,60,76]. These methods have been employed recently to provide insights into the risk factors and drivers of zoonotic pathogen emergence [77,78,79,80,81]. Nonetheless, history has shown that sustainable vector control programs are the most effective method in controlling mosquito populations [82], however their success hinges on continuous governmental financial support as well as buy-in and enforcement at the community level.

## 7. Conclusions and Future Prospects

ROCV is an emerging flavivirus responsible for the largest outbreak of arboviral encephalitis in Brazil. Despite several pioneering studies in the mid-1970s to late 1980s that provided some clarity on the epidemiology and clinical presentation of ROCV infections, its transmission cycles are not fully understood. Extensive serologic and ecologic surveillance studies on ROCV have not been carried out since then and the shortage and paucity of data has hindered our appreciation of the burden that ROCV infections may have on public health systems across Brazil and beyond. ROCV circulates in several regions of the country, infecting wildlife as well as animals important to agricultural and peri-domestic activities. As shown in the sections above, it is likely that the virus circulates undetected and most infections with mild symptoms are either underreported or attributed to other common arboviruses, such as dengue. The abundance of its mosquito vectors (*Psorophora.* and *Aedes species*) and vertebrate and amplification hosts (*Zonotrichia capensis*) provide the required conditions for ROCV’s potential to emerge and become an urgent public health issue. Furthermore, this review reiterates the urgent need for the establishment of comprehensive surveillance networks that are geographically broad, encompass hotspots of biodiversity as well as human cohorts, and are well integrated with appropriate modeling techniques that can mitigate the threat posed by emerging zoonotic and resurging arboviruses. It is essential that sound public health policy to contain and control zoonotic pathogens must also invest in the development of more accurate diagnostic assays using new and affordable technologies and effective vaccine or antiviral countermeasures.

## Figures and Tables

**Table 1:** Documented circulation of ROCV among humans.

Year	State	# of Cases	Diagnostic Tests Performed	Reference
1974–1977	São Paulo	>1000	Blood and serology (HI, CF)	[2,3,7]
1978–1983	São Paulo	4	Blood and serology (HI)	[58]
1984	Bahia	1	Blood and serology (neutralization test, HI)	[60]
1987	São Paulo	2	Blood and serology (neutralization test, MAC-ELISA)	[15]
1990	São Paulo	6	Blood and serology (neutralization test, HI)	[59]
1995	Bahia	8	Blood and serology (neutralization test, HI, MAC-ELISA)	[14]
2012–2013	Goiás	2	Blood, molecular (RT-PCR)	[9]

Abbreviations: HI—hemagglutination inhibition, CF—complement fixation, RT-PCR—reverse transcription polymerase chain reaction, MAC ELISA—immunoglobulin M enzyme-linked immunosorbent assay.

## Data Availability

Not applicable.
